# A fully automatic multichannel neural spike sorting algorithm with spike reduction and positional feature

**DOI:** 10.1088/1741-2552/ad647d

**Published:** 2024-08-05

**Authors:** Zeinab Mohammadi, Daniel J Denman, Achim Klug, Tim C Lei

**Affiliations:** 1 Department of Electrical Engineering, University of Colorado Denver, Denver, CO, United States of America; 2 Department of Physiology and Biophysics, University of Colorado Anschutz Medical Campus, Aurora, CO, United States of America

**Keywords:** spike sorting, neural signal processing, brain computer interface

## Abstract

*Objective*: The sorting of neural spike data recorded by multichannel and high channel neural probes such as Neuropixels, especially in real-time, remains a significant technical challenge. Most neural spike sorting algorithms focus on sorting neural spikes post-hoc for high sorting accuracy—but reducing the processing delay for fast sorting, potentially even live sorting, is generally not possible with these algorithms. *Approach*: Here we report our Graph nEtwork Multichannel sorting (GEMsort) algorithm, which is largely based on graph network, to allow rapid neural spike sorting for multiple neural recording channels. This was accomplished by two innovations: In GEMsort, duplicated neural spikes recorded from multiple channels were eliminated from duplicate channels by only selecting the highest amplitude neural spike in any channel for subsequent processing. In addition, the channel from which the representative neural spike was recorded was used as an additional feature to differentiate between neural spikes recorded from different neurons having similar temporal features. *Main results*: Synthetic and experimentally recorded multichannel neural recordings were used to evaluate the sorting performance of GEMsort. The sorting results of GEMsort were also compared with two other state-of-the-art sorting algorithms (Kilosort and Mountainsort) in sorting time and sorting agreements. *Significance*: GEMsort allows rapidly sort neural spikes and is highly suitable to be implemented with digital circuitry for high processing speed and channel scalability.

## 1. Introduction

Neurons in the brain fire action potentials, or spikes, which are then processed by multiple hierarchical neuronal circuits to extract biologically relevant information, thereby forming the basis for neural computation. It is therefore not surprising that many experimental approaches in neuroscience include the recording of trains of action potentials *in vivo,* in some cases even from awake and behaving animals. Such recordings are done by placing a recording channel of a neural probe or electrode in close vicinity to neurons in the extracellular space (=extracellular neural recording), and recording trains of action potentials under various experimental conditions (Rieke *et al* 1997, Vogels *et al* 2005, Barnett *et al* 2016, Buzsáki *et al* 2017, Rübel *et al* 2022). One challenge with such recordings is that under many recording conditions, an electrode can pick up neural spikes from several nearby neurons, resulting in so-called ‘multi-unit’ activity (MUA) in the recording trace (Humphrey and Schmidt 1990, Williams 2007, Pillow *et al* 2013, Cuevas 2014, Lee *et al* 2021). Spike sorting algorithms are then used to separate this MUA into several sets of ‘single-unit’ activities (SUA), each of which represents the action potential firing pattern of a single neuron (Lewicki 1998, Rey *et al* 2015, Magland *et al* 2020, Petersen *et al* 2021).

Over the years, many neural spike sorting algorithms have been developed. Many are focused on sorting neural spikes that were pre-recorded on a single recording channel (Lewicki 1998, Rey *et al* 2015). For instance, several unsupervised classification algorithms, such as K-means, Yass, DBScan and WaveClus, have been applied to sort neural spikes based on the classification of the temporal profiles of the pre-recorded neural spikes (Quiroga 2004, Yger *et al* 2018, Wang *et al* 2019, Lee *et al* 2020). Advanced signal processing techniques using discrete wavelet (Letelier and Weber 2000) and continuous wavelet-based approaches (Soleymankhani and Shalchyan 2021) have been applied in spike sorting. Wavelet packets can also be combined with Shannon mutual information and local discriminant basis to construct an effective cost function to improve spike sorting accuracies (Hulata *et al* 2002). On the other hand, independent component analysis (ICA) has been used to sort neural spikes recorded from microelectrodes with sparsely spaced but numerous electrodes (Leibig *et al* 2016, Buccino *et al* 2018). Additionally, classical statistical models including Gaussian mixture models (Souza *et al* 2019) and hidden Markov models (Herbst *et al* 2008) have been applied to more accurately sort neural spikes with low signal-to-noise ratios. In recent years, big data, machine learning, and deep learning approaches have also been used to learn the features of neural spikes to provide better sorting outcomes (Carlson and Carin 2019). Convolutional neural networks have been applied to differentiate different neural spikes in a non-linear manner (Li *et al* 2020, Wang *et al* 2023). Contextual machine learning approaches (SpikeDeeptector, Saif-Ur-Rehman *et al* 2019) and contractive autoencoders (Radmanesh *et al* 2022) have been used to classify neural spikes from noisy data with improved sorting accuracies by learning temporal and spatial patterns. On the hardware end, tensor processing units (TPUs) can also be used to accelerate computational speed to obtain inferences from machine learning models, enabling edge or off-grid computing (Rokai *et al* 2023). On the other hand, this plethora of algorithms mainly focuses on improving sorting accuracy and reducing the need for human intervention such as specifying the number of neuronal groups.

However, a much larger gap remains: The need for sorting algorithms which substantially reduce the requirement for computational processing power and system memory storage, while simultaneously achieving rapid neural spike sorting. Such an algorithm could be used to sort neural spikes in real-time, while the recorded data are streamed into the computer. The need for such an algorithm has increased significantly in recent years, as there has been a sustained effort to develop high density neural probes with hundreds of recording channels such as the Neuropixels probes (Jun *et al* 2017, Steinmetz *et al* 2018, Steinmetz *et al* 2021, Varol *et al* 2021). Because of the enormous amount of data recorded, new spike sorting algorithms have been developed to address the highly increased spike sorting needs (Pachitariu *et al* 2016, 2023, Rossant *et al* 2016, Chung *et al* 2017, Hilgen *et al* 2017, Boussard *et al* 2021, Paulk *et al* 2022). However, similarly to previous single recording channel algorithms, these algorithms use forms of global minimization which are required to be performed off-line and post-hoc, with significant computational power, and with relatively long computing time to arrive at the sorting outcomes. Thus, live spike sorting is not possible even with these algorithms.

Sorting neural spikes and decoding neural information in real-time may open new research paradigms to study neural circuits, for example by modulating neural activity in a closed-loop feedback manner to alter behavioral responses. To accomplish this, sorting neural spikes in real-time is an important step which allows for the extraction of neuronal information on the fly. This remains a significant technical challenge, especially when handling the large amount of data recorded from high-density multi-channel probes is involved (Buccino *et al* 2022). Compared to sorting pre-recorded neural spikes, real-time spike sorting requires the production of sorting outcomes with very short processing delay (< 10 ms) for closed-loop neuronal feedback applications. Therefore, global minimization approaches, by definition, cannot result in rapid sorting outcomes. Desirably, the algorithm should only require light-weight computations as well as limited use of memory storage to allow potential hardware miniaturization, which is especially important for the future design of devices such as neural implant. To that end, there have been sustained efforts to develop real-time neural spike sorting algorithms and systems. Many of these real-time spike sorting implementations are based on template matching, where spike templates are calculated by a tethered computer through a short training period and the learned spike templates are then loaded to custom hardware for spike matching (Jun *et al* 2017, Park *et al* 2017, Wang *et al* 2019).

**Figure 1.**
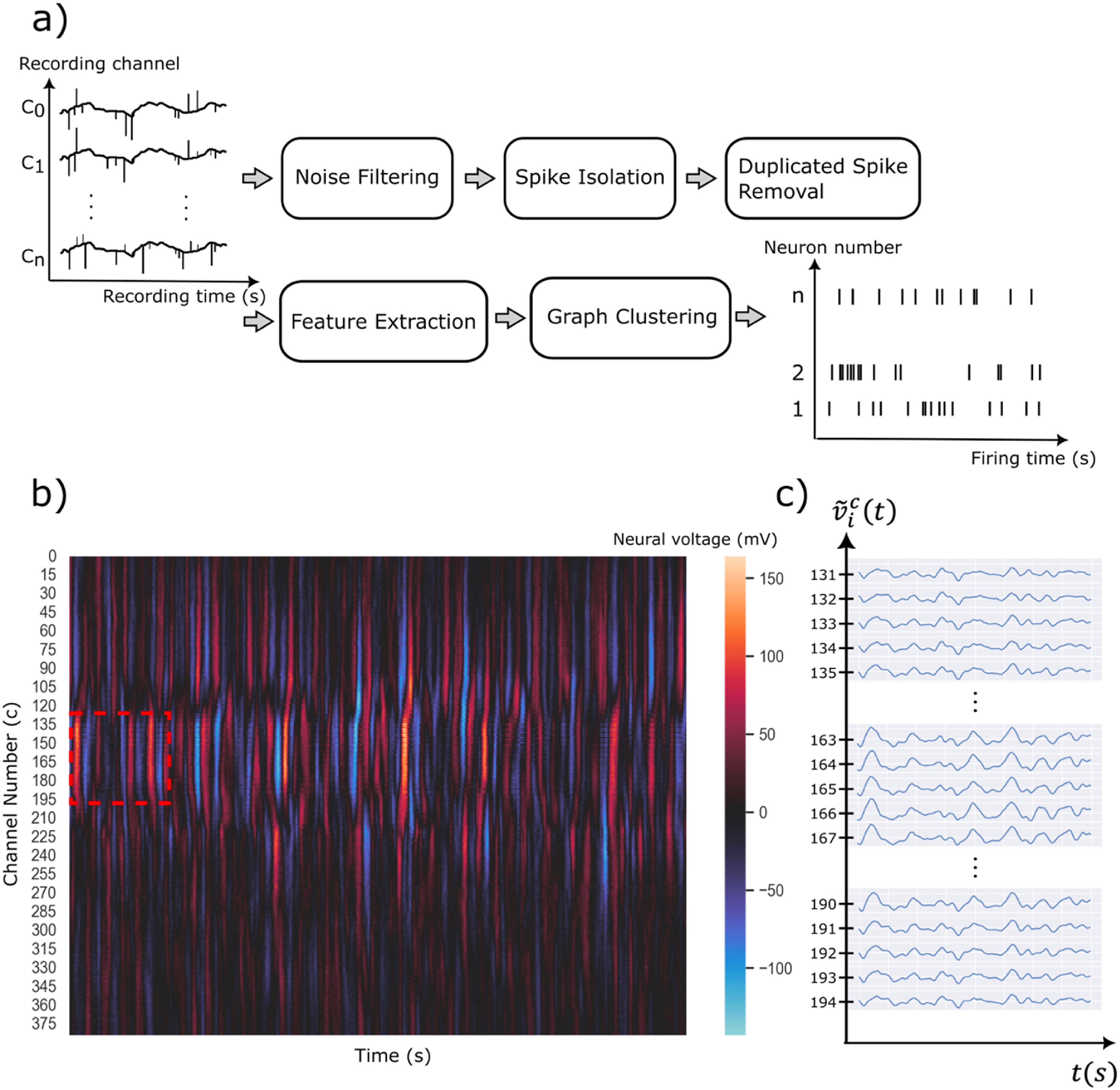
(a) The processing pipeline of the GEMsort spike sorting algorithm which processes raw multi-channel extracellular voltages to individual neural spike firing sequences (raster plot); (b) Intensity plot of extracellular neural voltage measured by a Neuropixels multi-channel probe recorded near the visual cortical region of a C57BL\6 J mouse; (c) The expanded voltage plot showing selected channels near the center and the two ends of the red dotted box of (b). It is obvious that duplicated spikes were recorded by adjacent channels.

In this manuscript, we propose a new method—Graph nEtwork Multichannel (GEMsort)—which uses a duplicated spikes data reduction approach to reduce the large amount of neural data, minimizing the required calculation without loss of sorting accuracy. The signal processing flow of GEMsort is illustrated in figure 1. In addition, GEMsort takes advantage of the graph network approach of using nodes and edges to form clusters to dynamically learn the neural spike features. With this graph approach, GEMsort is an inherently lightweight algorithm which can provide rapid neural spike sorting and can potentially be developed with digital hardware for a real-time spike sorting system.

## 2. Results

**Figure 2.**
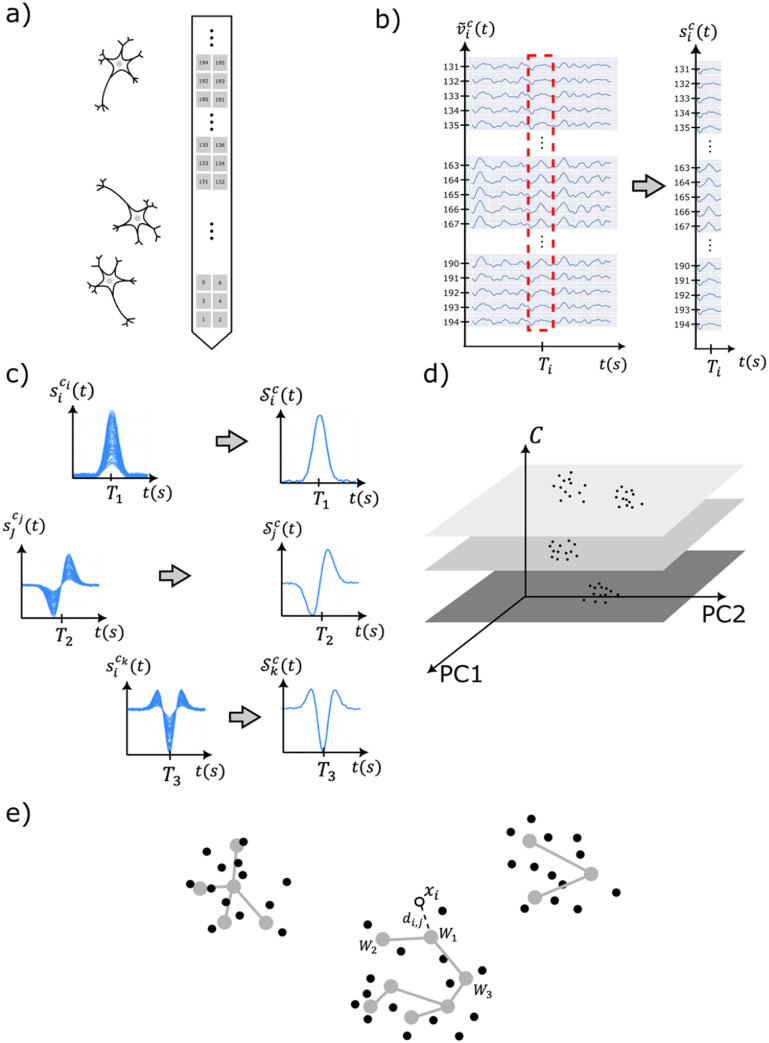
(a) An illustration showing a closely spaced multichannel probe for measuring extracellular firings of neurons; (b) Extracellular neural voltage $v_i^c\left( t \right)$ measured from a multichannel probe with channel number $c$ being isolated with a threshold value ${v_{{\text{th}}}}$ as $s_i^c\left( t \right)$; (c) multichannel isolated neural voltages contain duplicated neural spikes and the neural spike with the highest peak voltage was selected as representing neural spikes $S_j^C\left( t \right)$; (d) Features of the representing neural spikes $S_j^C\left( t \right)$ was extracted and projected on the feature space with the channel number *c* used as an additional feature to separate neurons measured from different neural probe channels; (e) Clusters connected with nodes (grey dots) and edges (grey edges) was used in the feature space to learn the neuron cluster distribution. An incoming neural spike can be classified to the closest neuron clusters based on the Euclidian distance ${d_{i,j}}$ between the incoming spike and the nodes. The closest node (*W_1_*) and the adjacent nodes (*W_2_* and *W_3_*) can move closer to the incoming neural spike ${x_i}$ for adaptability and learning.

The GEMsort algorithm was implemented using Python version 3.7 programming language and was run on a 3.4 GHz i7-4770 desktop computer with no use of GPU computation. Several sets of multichannel neural extracellular recording data were used to evaluate the performance of the GEMsort algorithm. The first set of data was a 16-channel synthetic neural data uses to demonstrate the sorting capabilities and accuracies of GEMsort under various physiology recording conditions. The second set of data was a pre-recorded electrophysiology neural recording measured with a Neuropixels probe and the data set was used to compare the sorting performance between GEMsort and Kilosort version 2.5 (Steinmetz *et al* 2021). This data set is available at https://ndownloader.figshare.com/files/37854918. The third set of data was another Neuropixels data obtained from (Pachitariu *et al* 2023) and this data set was used to compare the sorting performance between GEMsort and Mountainsort version 5. Figure 2 illustrates how the neural spikes measured by a multi-channel probe were transformed in the vector space using GEMsort for rapid spike sorting.

### 2.1. Performance evaluation using a synthetic 16-channel neural dataset

#### 2.1.1. Generation of the synthetic spike data

**Figure 3.**
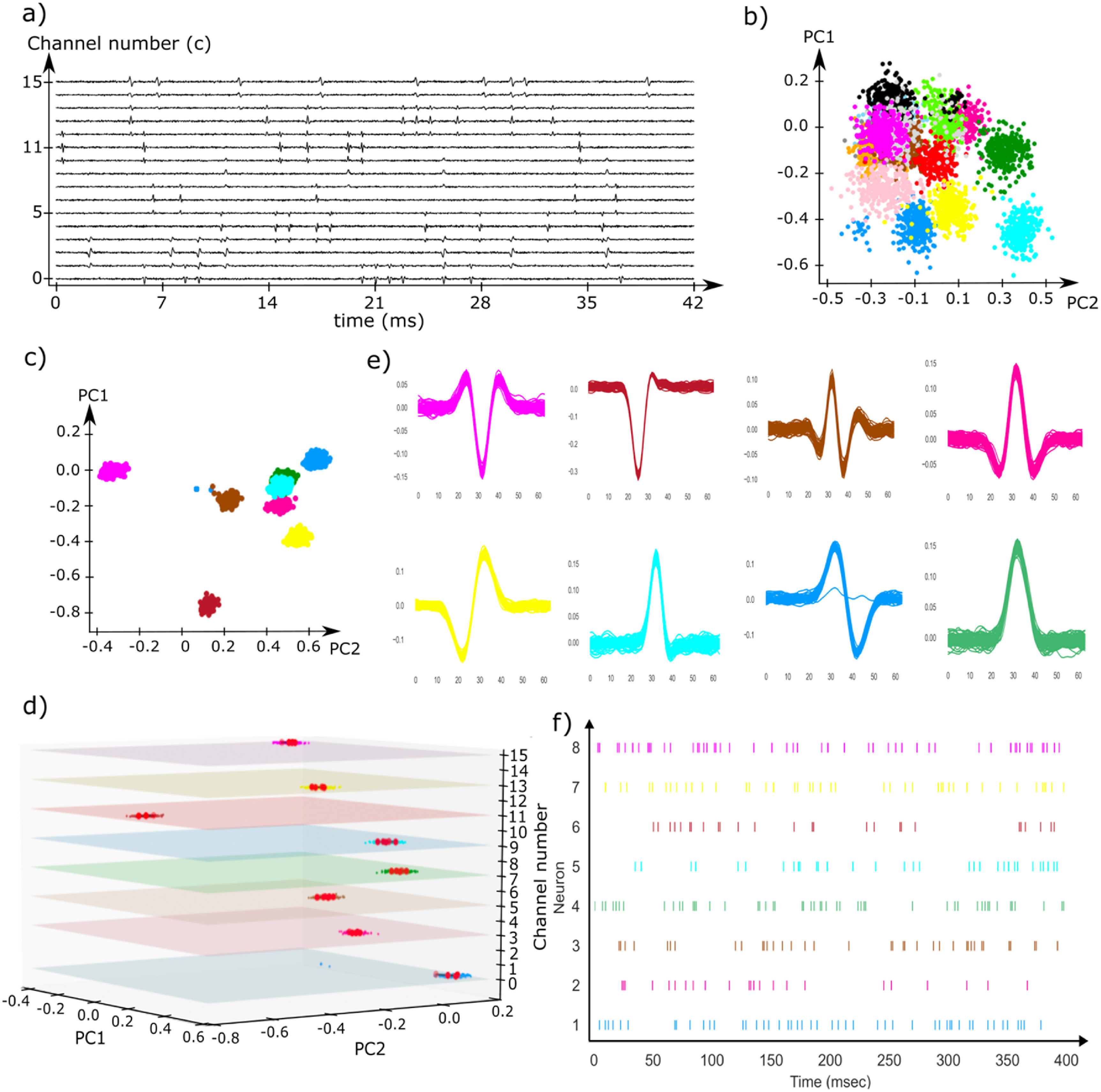
(a) Simulated neural voltages of 16 neural recording channels mimicking neuronal firing of 8 neurons; (b) Feature representation of all neural spikes measured by the 16 recording channels without elimination of duplicated neural spikes. Significant cluster overlap can be observed due to the artificial noise added to the neural spikes; (c) Feature representation of only the representing neural spikes after duplicated neural spike elimination. Neuronal clusters were more separated from one another despite there are still some clustering overlaps (red, cyan and green) due to similar spike profiles; (d) The overlapping clusters can be separated by adding the channel position number as an additional feature to separate neural spikes with similar temporal pulse shapes but the firing neurons were distributed at different channels; (e) Temporal pulse shapes of the 8 sorted clusters illustrates the correct sorting of neural spikes. (f) The neural firing activities (raster plot) of 8 neurons can be obtained by the firing times of the sorted spikes.

Multichannel synthetic neural data was useful to evaluate the performance of the GEMsort algorithm due to the flexibility of adjusting the parameters of the dataset to achieve certain testing conditions while maintaining reasonable levels of complexity and realism. To imitate spontaneous neuronal background firing which generally follows Poison statistics, the extracellular spike sequences of the 16 channels were generated with a Poisson generator. User selected firing rates ${f_{{\text{rate}}}}$ ($1 &lt; {f_{{\text{rate}}}} &lt; 200$) were used to constrain the Poisson generator to generate firing sequences for the neurons for specific firing rates. Eight spiking neurons were then placed in the immediate vicinity of channel 0, 3, 5, 7, 9, 11, 13 and 15, causing these channels to receive the strongest neural signals from their respective closest neurons. Neural spikes were generated using different wavelet shapes to mimic various temporal profiles fired by different neurons. The temporal profiles of the neural spikes used for the 8 neurons included Gaussian, Richer, Biorthogonal, and Daubechies wavelets (Lee *et al* 2019). Gaussian noises with a variance of 0.02 were added to the synthetic neural spike data to simulate noise contamination caused by the recording environment or other sources. Signal reduction due to distance ($r$) between the neurons and the recording channels were modeled based on a parabolic voltage potential ${V_e}\left( r \right) = 1/4\pi \sigma r$, where the extracellular fluid conduction $\sigma = 0.35$ [36]. Figure 3(a) shows an example of synthetic neural voltages plotting the 16 channels to mimic a multi-channel neural recording.

Special conditions were also evaluated using synthetic neural spikes to test whether GEMsort can handle some extreme neural recording conditions. Despite these firing patterns are not realistic, these firing patterns, however, can be used as benchmarks to test GEMsort whether it can still handle the sort properly under these extreme conditions. If GEMsort can handle these extreme firing patterns well, it can imply that GEMsort should work well for typical firing conditions. For this reason, three different synthetic neural spiking datasets were created. The first synthetic neural spiking dataset mimics the scenario when two neurons fired exactly at the same time but with different spike temporal shapes. The second dataset simulates the scenario when the neural spikes fired from two different neurons having similar temporal spike shapes. Third a comparison dataset was also created to demonstrate the most common scenario when the neurons are randomly placed along the neural probes and unrestricted on the firing time and spike temporal shapes.

#### 2.1.2. Sorting synthetic neural spikes with GEMsort

The 16-channel synthetic neural voltages to mimic neural spike firing by 8 neurons were sent to the GEMsort algorithm through the processing pipeline for neural spike sorting. As discussed in the methods section, for closely spaced multichannel neural probes, the same neural spikes can be picked up by several adjacent recording channels, resulting in the same spike being observed multiple times. Figure 3(b)) shows the feature representation of all neural spikes recorded from the 16 recording channels without any duplicated neural spike elimination. It is obvious that the neural spikes were overlapping with one another. The spreading and overlap of the clusters were due to artificial noise being added to the neural spikes and these overlaps make spike classification difficult. In addition, if all the neural spikes were sorted without elimination, the same neural spikes were sorted multiple times, leading to incorrect spike rates for sorting outcomes, as well as taking longer time and computational resources to process. In contrast, figure 3(c) shows the feature representation of only the representing neural spikes. With the elimination of the duplicated neural spikes and only using the strongest neural spikes for the sort, the clusters were more separated from one another, making cluster classification much easier and more accurate. Another important benefit of only sorting the representing neural spikes is that it avoids over-sorting the duplicated spikes such that accurate firing rates can be calculated for further signal analysis. However, there are still clusters (red, cyan and green) which closely overlap in figure 3(c) since the neural spikes of these clusters had similar temporal profiles, and therefore similar features. GEMsort solved this issue using the recording channel number as an additional feature to separate neural spikes with similar temporal profiles but fired from different neurons at different locations on the neural probe. Figure 3(d) is the 3D feature plot using the channel number as a feature to separate overlapping clusters. With the additional channel feature, the overlapping clusters were further separated to allow proper neural spike classification. Over the total 2242 isolated neural spikes, without the channel feature, the sorting accuracy was only 79.8% due to the significant cluster overlap. With the additional channel feature, the sorting accuracy was improved to 93.0%, and the sorted neural spikes of the 8 cluster groups were plotted in figure 3(e)). The remaining misclassification was likely due to closely spaced neurons firing at similar time intervals, resulting in temporal profiles that cannot fall into any of the cluster groups leading to misclassification, or simply noise contamination. Since the spike time ${T_i}$ was associated with the representing neural spikes $\mathcal{S}_i^c\left( t \right)$,once the neural spikes were sorted, the sorting outcomes could use ${T_i}$ to construct a raster plot. Figure 3(f) is an example raster plot generated with the sorting outcomes of this specific simulated neural voltages.

**Figure 4.**
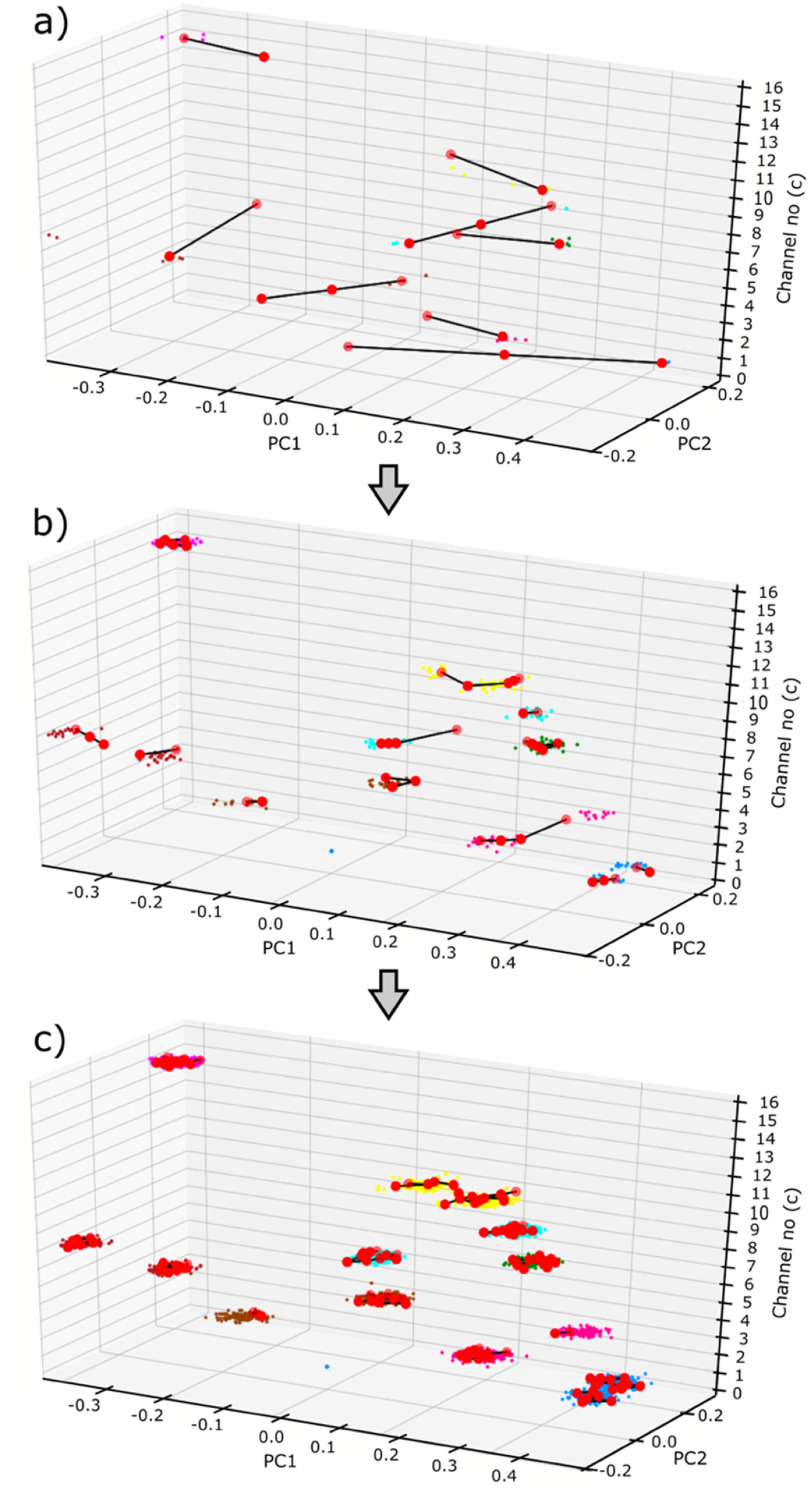
Neural spike classification in the feature space. (a) Early classification process when only a few neural spikes streamed into the algorithm and early interconnecting nodes were formed to learn the neuronal clustering distribution; (b) As more neural spikes were streamed into the algorithm, more nodes were generated to form larger clusters and the neural spikes started to be correctly sorted; (c) Through node movements and edge connections or eliminations, stable neuron clusters can be formed to provide rapid neural spikes sorting. If feature distribution of the neuron clusters changed, the nodes and edges can move to conform to the new feature distribution to allow continue proper neural spike sorting. This classification can be better visualized with the video in the supplementary information.

Figure 4 illustrates the classification process using a graph network. In the beginning of the sort, as shown in figure 4(a), only a small number of neural spikes were streamed into the feature space, and a few nodes were generated to cover these initial spikes to form clusters. As more neural spikes were streamed into the feature space and analyzed by the algorithm, more nodes were generated and interconnected to form larger clusters as shown in figure 4(b), allowing the sort to be more accurate as the cluster stabilized. At the end of the sort, as shown in figure 4(c), the clusters were fully stabilized to cover the neural spikes for accurate sort. Note that these clusters could continue to adapt, should the temporal shapes of the neural spikes change, allowing the algorithms to learn the neural spike distribution on the fly to accustom for changes or noise contaminations. This self-learning feature makes GEMsort particularly useful for sorting neural spikes during long-term neural recordings. It is also important to mention that as the streamed neural spikes were analyzed and learned by the graph clusters, the neural spikes were not further retained in the system memory, allowing GEMsort to be particularly nimble and not requiring extensive system hardware. A video demonstrating the classification process of the graph network classification approach can be found in the supplementary information, illustrating how graph networks can learn and adapt to neural features and graph clustering on the fly while neural spikes were streamed into the processing pipeline.

#### 2.1.3. Sorting simultaneous firing and similar spike shapes

**Figure 5.**
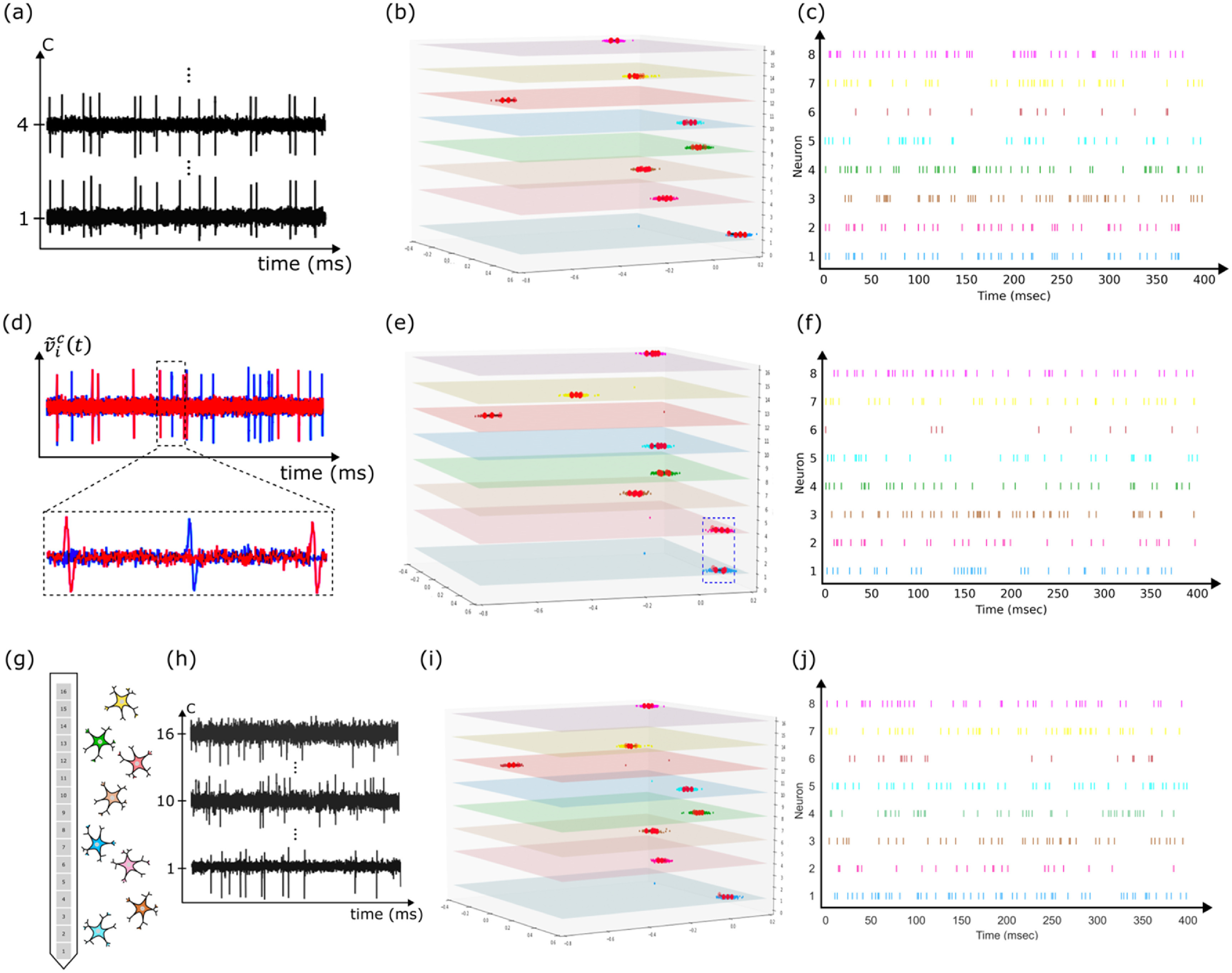
(a) Simultaneous spike firing of 2 neurons at channel 1 and 4; (b) Neural spike clusters of the 8 neurons well separated by the shape and channel features although the two cells fired at the same time; (c) The resulting raster plot showing neuron 1 and 2 fired at the same time; (d) Another synthetic spike sequences in which 2 neurons fired neural spikes with similar spike shapes; (e) The 2 neurons firing neural spikes with similar spike shapes clustered at the similar region (dotted box) were separated by the channel feature; (f) The reconstructed raster plot correctly classified the neural spikes to their neurons; (g) and (h) An unrestricted neural spike sequence served a control comparison; (i) Separated clusters in the feature space; (j) Raster plots created from the sorting outcomes.

Figure 5 demonstrates the robustness of GEMsort of handling atypical neural firing sequences. Synthetic neural spikes fired by 8 neurons distributed along a 16-channel neural probe. Among these 8 neurons, 2 neurons located on channel 1 and 4 specified designed to fire at the same time to allow testing GEMsort’s capability to differentiate neural spikes fired at the same time, as shown in figure 5(a). Since GEMsort does not use firing time as a feature for spike classification and the neural spike shapes fired by these two neurons are different, the clusters of neurons 1 and 2 were separated far apart from one another in the feature space. Therefore, simultaneous neural spikes can be correctly classified and have been correctly sorted to either neuron 1 or 2 based on the features. Figure 5(c) is the resulting raster plot generated from the sorting outcomes, and the firing sequences of neuron 1 and 2 can be correctly recreated to have the same firing times. Likewise, neural spikes having similar spike shapes can also create a challenge for sorting algorithms. GEMsort was evaluated using a synthetic neural spike sequence in which 2 of the 8 neurons fired with similar spike shapes, as shown in figure 5(d). Despite the neural clusters of the 2 neurons being in similar regions, as shown in figure 5(e), the channel feature helped separate these two clusters (the dotted box highlighting these two clusters being separated by the channel feature), resulting in a correct raster plot outcome (figure 5(f)). Finally, a comparison spike dataset with no specific constraint on neuron location, spike firing timing and spike shapes (figures 5(g) and (h)) were sorted to serve as a control for comparing the sorting accuracy to the previous two cases. The clusters in the feature space (figure 5(i)) reflecting the clustering situations for common neural spike recordings in which the clusters are reasonably separated to allow generating the raster plot (figure 5(j)) which can be used for further neural signal processing, such as neural signal decoding. Table 1 shows the sorting accuracies of the three datasets and the sorting accuracies of all the three scenarios are above 92%, indicating that GEMsort is robust in sorting neural spikes with simultaneous firing times and/or with similar spike shapes. Table 1 also highlights the importance of the channel feature to assist cluster separation for multichannel recordings. Without the use of the channel feature, clusters were overlapped due to similarity in spike temporal shapes, and these overlapping clusters resulted in low sorting accuracies of not higher than 65%. Table 2 listed the sorting accuracies of the synthetic data sets compared to the ground-truth labels and the sorts generally can achieve a high degree of accuracies of higher than 90%.

**Table 1.** Comparison of sorting accuracies with and without using the channel feature between three synthetic neural spike datasets. The first dataset has 2 of the 8 neurons fired at the same time but with different spike profiles; The second dataset has 2 of the 8 neurons having similar spike profiles; the last dataset is unrestricted of the firing time and the spike profiles to serve as a comparison control. The sorting accuracies for all three cases can reach above 92% with the channel feature used to assist in the cluster separation, while the sorting accuracies are significantly reduced without using the channel number as a feature.

	Sorting accuracy	
	Without channel feature	With channel feature
Simultaneous firing neurons	0.6431	0.9204
Similar spike shapes	0.5973	0.9456
Unrestricted spike sequences	0.6278	0.9482

**Table 2.** GEMsort sorting outcomes compared to the ground-truth labels of the neural spikes for the 4 synthetic 16 channel neural data sets.

Sorting accuracy	Cell 1	Cell 2	Cell 3	Cell 4	Cell 5	Cell 6	Cell 7	Cell 8
General 16 channel data (figure 3)	0.996	0.951	0.88	1	0.998	0.63	1	0.997
Simultaneously firing of two neurons (figure 5(a))	1	1	0.956	0.93	0.995	0.72	0.996	1
Similar spike profiles of two neurons (figure 5(d))	1	0.898	0.90	1	0.996	0.67	1	0.989
Comparison data (figure 5(e))	1	1	0.904	0.988	1	0.65	1	0.996

### 2.2. Sorting multichannel neural voltages measured by Neuropixels

#### 2.2.1. Neural data recorded by a Neuropixels probe

The GEMsort algorithm was also evaluated using prerecorded multichannel neural data measured by a Neuropixels probe. Neuropixels is a single shaft multi-channel neural probe consisting of 384 recording channels selectively measuring from 960 recording sites with a spacing between the recording sites of ∼20 *μ*m. For the neural data used in this paper comparing the sorting agreement between GEMsort and Kilosort (version 2.5), neural data were recorded by Neuropixels Phase 3A prototype probes (Jun *et al* 2017) in awake mice, following procedures described in detail elsewhere (Denman and Reid 2019). All animal procedures were approved by the Allen Institute for Brain Science institutional animal care and use committee. Mice in this study were male C57BL\6 J aged 60–182 d. Briefly, animals were first outfitted with a permanently attached head fixation device and habituated to the recording apparatus. On the day of recording, an approximately 2 × 2 mm cranial window was opened over the primary visual cortex, and a Neuropixels probe lowered through cortex, subiculum or hippocampus, and ventral thalamic structures. Probes were inserted with piezoelectric manipulators at 50–100 *µ*m^-^min to their target depth. Increased neural activity was elicited with visual stimuli, which consisted of full-field flicker, repeated presentation of natural images, and a repeated naturalistic movie clip. The data we used in this paper was measured from one mouse (*n* = 1). In order to demonstrate that GEMsort can be used as automatic sorting algorithm without need for user intervention, sorting results of GEMsort were compared to those of Mountainsort (version 5.0), which can be used as an ‘automatic’ multi-channel sorting algorithm and does not require signal curation, to calculate sorting agreement and processing time. The data set used in this comparison was a Neuropixels recording obtained from (Steinmetz *et al* 2019, Pachitariu *et al* 2023)

#### 2.2.2. Sorting comparison between GEMsort and Kilosort

A 200 s segment of Neuropixels neural recording data was sent to our Python GEMsort code and the Kilosort spike sorting routine (version 2.5) to compare sorting results. The sampling rate of the recording was 30 kHz which resulted in 6,000,000 data points for each recording channel. With 384 total recording channels, a total of 384x6,000,000 data points were processed by both algorithms. A Windows 10 desktop computer equipped with a i7-4770 CPU running at 3.40 GHz, a Nvidia Titan RTX GPU, and 24 GB of system memory were used for both algorithms. However, there is one computational difference. Since Kilosort can take advantage of GPU acceleration, GPU computation was used for Kilosort. On the other hand, since GEMsort was designed to minimize hardware requirements, no GPU acceleration was therefore used for GEMsort. Despite not using any GPU processing for GEMsort, the processing time for sorting 200 s of 384 recording channels was ∼279 s. In comparison, the processing time for Kilosort was ∼543 s, which was approximately two times longer than GEMsort. In addition, Kilosort requires manual curation to combine or split clusters, which is not required for GEMsort, and this manual processing time was subjective and therefore was not included.

**Figure 6.**
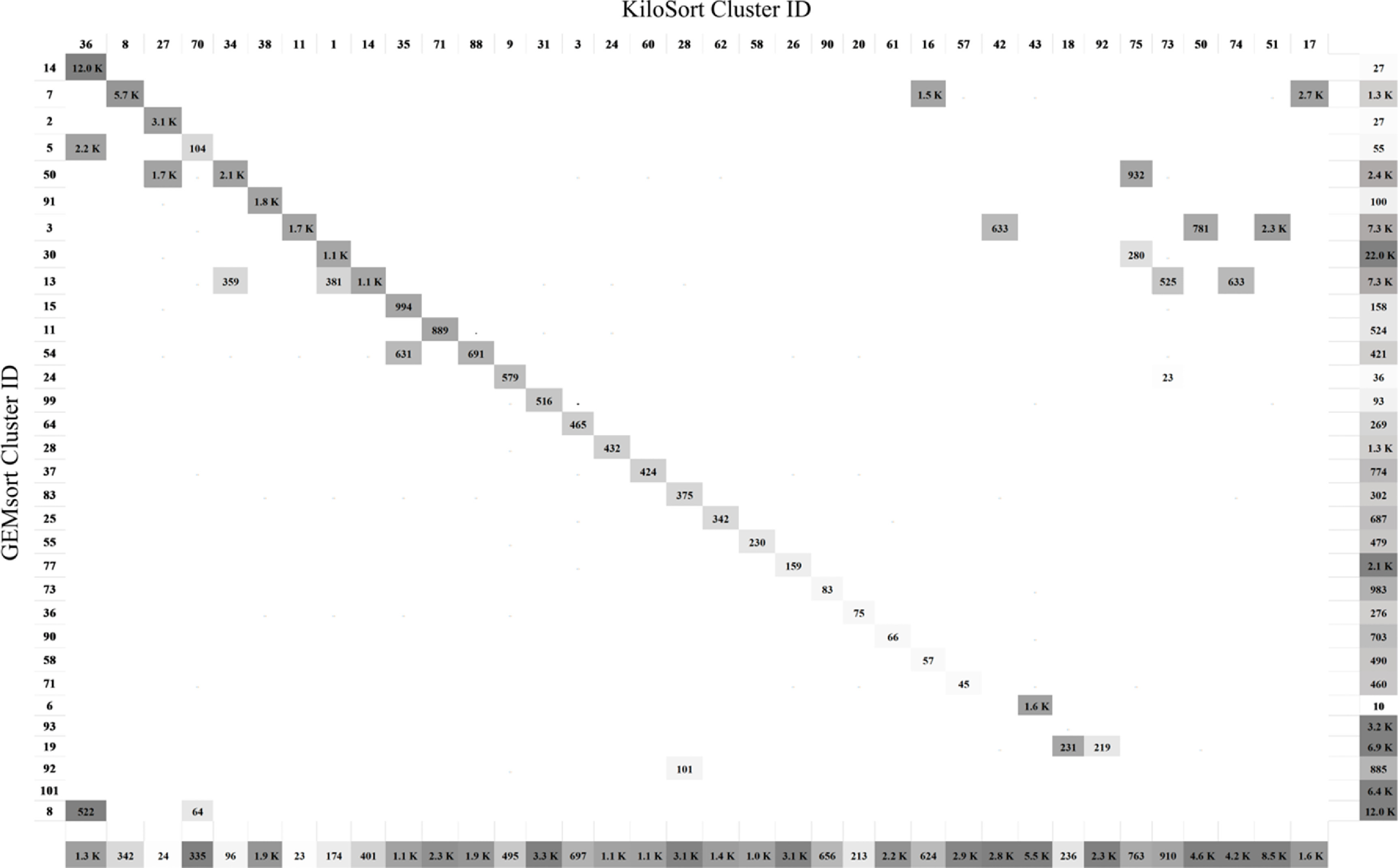
Confusion matrix comparison of sorting agreement between GEMsort and Kilosort (version 2.5) using Neuropixels neural recording. The left-vertical and the top-horizontal axes show the GEMsort cluster ID number and the Kilosort cluster ID number respectively. The square element within the confusion matrix represents the numbers of common spikes between the two sorting algorithms and a grey-scale intensity within the square element were used to indicate the matching percentage between the two sorting algorithms. The right-vertical axis shows the numbers of neural spikes and the percentages colored in grey-scale sorted by GEMsort, while the bottom-horizontal axis shows the same information sorted by Kilosort.

Figure 6 shows the confusion matrix for comparing the sorting outcomes between GEMsort and Kilosort. The left-vertical and the top-horizontal axes show the GEMsort cluster ID number and the Kilosort cluster ID number respectively. The square element within the confusion matrix represents the numbers of common spikes between the two sorting algorithms. A grey-scale intensity within the square element was used to indicate the matching percentage between the two sorting algorithms (the darker of element, the better the match). The elements of the bottom-horizontal axis show the numbers of neural spikes and the percentages (in grey-scale intensities) that could be sorted to the corresponding Kilosort clusters but could not be matched to any GEMsort clusters. Similarly, the right-vertical axis shows the opposite situation. For this Neuropixels recording clip, Kilosort detected 512 neuronal clusters before manual curation. After manual curation, 93 selected clusters remained based on visual inspection and labeling the clusters as valid, MUA, or noise contaminations, as well as merging or splitting the clusters. By comparison, GEMsort detected 106 neural clusters without the need for manual curation. Based on the sorting results, the GEMsort and Kilosort neuron IDs were respectively numbered from cluster 1–93 and from cluster 1–106.

The diagonal elements of figure 6 indicate matched sorting outcomes between the two algorithms. Besides these matched results, there are a few off-diagonal elements which indicate sorting disagreements between the two algorithms. These disagreements were mainly caused by one algorithm which considered the neural spikes to belong to the same neural cluster while the other algorithm considered them to belong to two different clusters. The neural spikes on the bottom-horizontal and right-vertical axes were considered to be neural spikes by one algorithm but considered to be noise or MUA by the other algorithm. In addition, we have measured the spike agreement between the two techniques. For neurons with more than 1000 spikes, the spike agreement between the two techniques was 63%. While for neurons with more than 100 spikes, the spike agreement was slightly reduced to 62%.

#### 2.2.3. Sorting comparison between GEMsort and Mountainsort

A public domain Neuropixels neural recording dataset (Steinmetz *et al* 2019, Pachitariu *et al* 2023) was used to compare the sorting agreement and sorting time between the GEMsort and Mountainsort (version 5). Besides comparing the sorting performances of the two algorithms, this comparison also used to demonstrate that GEMsort, like Mountainsort, is a fully automated sorting routines which does not require manual interventions by the users.

A 60 s segment of recording, which was equal to 1,800,000 data points, was used in the comparison. The Della cluster at Princeton University (Della computer cluster 2023) equipped with 128 GBs of system memory was used for this comparison. Despite the Della cluster being equipped with A100 GPUs, these were not used for both sorting algorithms. The SpikeInterface framework (Buccino *et al* 2020) was used to read the data for Mountainsort to analyze the neural spikes. In this evaluation, GEMsort needed 316.4 s while Mountainsort needed 2851.3 s to complete the sort. Besides the sorting time, the number of detected neurons, the number of detected spikes, the neuron temporal profile similarity, the number of spikes with similar firing times, and the sorting agreement in percentage are listed in table 3.

**Table 3.** Comparison of sorting performances between GEMsort and Mountainsort (version 5) using an Neuropixels recording (Pachitariu *et al* 2023) in several categories.

	Number of detected neurons	Number of detected spikes	Neuron temporal profile similarity	Spikes with similar firing times	Sorting agreement
Mountainsort5	197	109 667	70%	86 795	75.5%
GEMsort	215	106 977			

#### 2.2.4. Supplementary data analysis

Additional data analysis and statistical quantifications can be found in the supplementary information. GEMsort’s sorting performance, including sorting stability and sorting accuracy, was quantitatively analyzed as the data size increased (S1), as well as with the increase in noise contamination (S2). In addition, hyperparameter selections were analyzed, showing reasonably stable sorting results (S3), indicating that parameter selection within a reasonable range does not significantly affect sorting results. Simulated neural data generated from a 2D multichannel probe were also analyzed, demonstrating that GEMsort can handle 2D neural data structures as well as 1D neural data structures (S4). Furthermore, autocorrelograms of sorted neural spikes were analyzed, demonstrating dips within the refractory period (S5)—an indication of clean sorting.

## 3. Discussion

GEMsort was designed to sort neural spikes rapidly. Compared to many existing neural spike sorting algorithms which were designed to sort neural spikes post-hoc in an off-line manner, GEMsort uses a different approach to analyze the neural spike only once and estimate the neuronal clustering with a short processing time. In addition, GEMsort allows several of its processing steps, including the spike elimination and feature extraction modules, to be implemented using a parallel computation architecture. This modular capability allows scaling up the number of neural spikes to be processed without scarifying the processing time. This parallel design is particularly suited to be implemented by digital hardware.

All the parameters needed for the GEMsort algorithm are listed in table 4. More nodes were used for the Neuropixels data, but otherwise the same parameters were used to sort both the 16-channel synthetic neural data and the Neuropixels data. In addition, GEMsort does not require any manual intervention in the sorting. Not only can this avoid subjective sorting judgments, this feature can also be important for close-loop neural circuit control or live and real-time neural data analysis during an experiment, since there might not be time to perform manual processing, especially when an animal is under sedation and only limited amount of time is available to perform an experiment (Edward *et al* 2018).

**Table 4.** Parameters required for the GEMSort spike sorting algorithm. Typical parametric range and the parameter values used for the results demonstrated in this paper are listed in the first and second columns respectively.

Parameters	Typical ranges	Values used in this paper
Maximum node count (N)	3 to Max (it depends on the data and the recording area of the brain)	10–15 for every channel of the synthetic 16-channel MEA (it can be changed based on the Neural density of the recording area)
Edge pruning threshold (a_th_)	A fraction of total node count (e.g., half or one-third).	4–8 for the synthetic 16-channel MEA data
Number of initial nodes (N_int_)	2	2
Moving rate of W_1_ (e_s1_)	(0, 1)	0.8
Moving rates of W_j_ (e_nbr_)	(0, 1), ${{\text{e}}_{{\text{s1}}}} &gt; {{\text{e}}_{{\text{nbr}}}}$	0.001
Insert parameter reduce rate of w_e_ and ${{\text{W}}^{^{\prime}}_{\text{e}}}$ nodes (*α*)	(0, 1)	0.5
Insert parameter reduce rate of other nodes (*β*)	(0, 1)	0.01
Number of iterations before inserting a new node (*λ*)	5–50	10

In this paper, the sorting results between GEMsort and Kilosort were compared. This comparison was used mainly to evaluate whether GEMsort can reach the sorting performance of Kilosort in a simulated off-line sorting scenario. Despite a high degree of agreement between the two sorting algorithms, there remains some discrepancy between the Kilosort’s global optimization and GEMsort’s graph-based approach. While some difference in results were expected between the post-hoc designed approach of methods like Kilosort and GEMsort and the streaming approach of GEMsort, it is, however, important to further refinement of the algorithm and the possibility of adding subjective ground truth sorting comparisons to guide further improvements to the algorithm.

A potential extension of GEMsort includes identifying spatiotemporally overlapping neural spikes. GEMsort currently cannot correctly sort mixed neural spikes in situations where two nearby neurons fired at the same time, and considered these overlapping events either as outliers or misclassified them to a neuronal cluster. In Kilosort, a second processing pass was used to identify these overlapping neural spikes by matching them to mixed spike templates estimated from the initial processing pass. GEMsort could utilize the same idea by adding a final processing stage at the very end to match neural spikes that are not close to the neuron clusters to two mixed cluster groups.

It is worth mentioning that GEMsort was not designed to compete with existing spike sorting algorithms in sorting accuracy; rather, the design goal of GEMsort was to provide a rapid spike sorting platform that can deliver reasonable sorting agreements. Current existing spike sorting algorithms are typically designed to examine the entire neural recording and therefore have a global perspective to seek the most optimal sorting outcomes. In contrast, GEMsort can only learn from past neural spikes and therefore lacks the ability to examine neural spikes in the future. Therefore, existing neural spike sorting algorithms are most suitable for applications requiring the highest sorting accuracies, while GEMsort was designed to provide rapid, or even real-time, spike sorting capability for applications requiring immediate sorting outcomes.

It is well known that neuronal firing and neural signal encoding in the brain has significant firing variability. Neuronal responses to the same stimulus typically vary to some degree on a trial by trial basis. Therefore, firing rate averages acquired from many trials are typically used to study neuronal circuits. However, the brain uses coding redundancy to combat these fluctuations and uses ensemble coding or other more complex encoding schemes. Therefore, for real-time neural information decoding, small spike sorting fluctuations due to occasional spike overlaps may not be a significant factor in neuronal decoding interpretation. The lack of real-time neural signal decoding tools is one of the reasons why single trial real-time neural signal has yet to be used for neuroscience studies, and future neural signal processing should be further developed to solve the problem. To that end, GEMsort may be allow extraction of real-time SUA from high-density neural electrodes to allow for novel exploration of neural circuits.

## 4. Conclusion

Through the combination of duplicated spike elimination, positional feature and graph network clustering, GEMsort demonstrated that it has the potential to be used to sort neural spikes measured in real-time. The hardware requirement for the GEMsort algorithm is also significantly less than some of the existing neural spike sorting routines. These technical benefits make GEMsort viable choice as the frontend neural spike sorting processing unit to extract SUAs for real-time neural information decoding.

### 4.1. Methods

In this section, the GEM neural spike sorting method for sorting neural spikes measured from a densely spaced multichannel probe is discussed. The GEM algorithm was designed to learn neural spike clustering features dynamically and on the fly and to classify the neural spikes with almost instantaneous sorting outcomes. With this design philosophy in mind, GEMsort can potentially be used to obtain SUAs during neural recording. This information can be used to further decode neural information and may even be extended to modulate neuronal activity in a closed-loop manner. Compared to other spike sorting techniques which minimize the difference between the entire measured data and the reconstructed spike patterns, GEM processes the measured neural spikes only once without the need to retain neural spikes in the system memory, thus significantly saving memory and computational resources. This is largely due to the fact that GEMsort uses nodes and edges to learn neural spike clustering features and the clusters formed by these nodes and edges are used as a surrogate to represent the clustering relations. Another advantage of using the graph approach is that the clusters formed by these nodes and edges can be adaptively adjusted during the sorting process to compensate for changes in waveform and action potential shape during long duration recordings where the electrode or brain tissue may shift.

Figure 1(a) shows the processing pipeline of GEMsort. Neural voltages recorded from a Neuropixels multichannel neural probe (figure 1(b)) were first sent to a noise filter to remove unwanted noise and channel crosstalk contamination. Neural spikes were then isolated from the neural voltages measured from the multiple recording channels by using a voltage threshold. Figure 1(c) is a zoom-in plot for neural spikes recorded by neighboring channels, and it is obvious that in some cases duplicate spikes were recorded by adjacent channels. From these data, the neural spike with the highest signal height was identified to represent all the duplicate neural spikes arriving simultaneously to neighboring channels in the closed-spaced recording situation. Note that this approach is different from what other neural spike sorting algorithms do. Features of the representing neural spikes were then extracted using dimensional reduction algorithms with principal component analysis (PCA) in the current implementation. Finally, GEMsort utilizes graph clustering based on the EGNG algorithm in which nodes and edges were used to form clusters to represent the neuronal clusters. Another innovation for GEMsort is that the channel from which the representing neuron spikes were measured was also used as a spike feature to allow multichannel sorting, even when the spike features were similar for neurons residing at different locations. The cluster number, spike time and the channel number were estimated as sorting outcomes, which can then be used to construct spike timing histograms for each of the individual neurons.

Figure 2(a) is a schematic diagram illustrating a multichannel neural probe measuring neural firing activities from neurons in the vicinity of the probe. Based on this configuration, the algorithmic details of the GEMsort algorithm will be discussed in the following sections.

### 4.2. Noise-elimination and neural spike isolation

A second-order high-pass Butterworth filter was employed to remove low-frequency fluctuations and Local field potentials (LFP) from the multichannel neural recordings, and a 3 dB cut-off frequency of 300 Hz was used to filter the recorded neural voltages. Noise filtered from the neural voltages included electrical and biological artifacts, electrode motion and tissue movements.

A Local common average referencing (L-CAR) filter was then applied to the recorded neural voltages to remove correlated noise that is common across adjacent channels (Ludwig *et al* 2009, Xinyu *et al* 2017). The purpose of L-CARS was to remove large-area common noise contaminating the recording channel to allow better spike isolation. Suppose the neural voltage recorded from the ${c^{{\text{th}}}}$ channel of the multielectrode probe at time $t{\text{ }}$is ${v_i}\left( t \right)$, an averaged voltage ${\bar v^c}_{{\text{noise}}}\left( t \right)$ from a group of channels away from the ${c^{{\text{th}}}}$ channel was used to approximate the background noise. ${N_{{\text{far}}}}$ and ${N_{{\text{near}}}}$ (${N_{{\text{far}}}} &gt; {N_{{\text{near}}}}$) are the farthest and nearest numbers of channels to be included to generate this noise average ${\bar v^c}_{{\text{noise}}}\left( t \right)$. The two channel numbers can be set by investigators depending on the distance between the recording sites of the MEA electrode, the intended goal of the recording, and the level of the noise in the neural signal. The noise average ${\bar v^c}_{{\text{noise}}}\left( t \right)$ can be expressed mathematically as

\begin{align*}{\bar v^c}_{noise}\left( t \right) = \frac{1}{n}\left\{ {\mathop \sum \limits_{k = c - {N_{near}}/2}^{c - {N_{far}}/2} {v_k}\left( t \right) + \mathop \sum \limits_{k = c + {N_{near}}/2}^{c + {N_{far}}/2} {v_k}\left( t \right)} \right\}\end{align*}

where $n$ is the total number of channels included. The L-CARS filtered voltage for channel $c{\text{ }}$can be calculated by subtracting this average in which $\tilde v_i^c\left( t \right)= v_i^c\left( t \right) - {\bar v^c}_{{\text{noise}}}\left( t \right)$

After noise was removed from the multichannel neural recording, a threshold ${v_{{\text{th}}}}$ was used to differentiate and isolate neural spikes from the filtered neural voltage $\tilde v_i^c\left( t \right)$, as shown in figure 2(b). The threshold ${v_{{\text{th}}}}$ can be automatically determined based on the standard deviation $\sigma $ of the multichannel neural recording or manually determined based on custom criteria. In this paper, the threshold was set to be 5 times the signal standard deviation of each channel ($v_{{\text{th}}}^c = 5{\sigma _c}$ and ${\sigma _c} = \overline {\tilde v_i^c\left( t \right)} /0.67$). A neural spike was detected from the filtered neural voltage $\tilde v_i^c\left( t \right)$ if the signal was higher than the threshold ${v_{{\text{th}}}}$ and the isolated neural spike was denoted as $s_i^c\left( t \right)$ where $i$ is the isolated spike index and $c{\text{ }}$is the recording channel from which the neural spike was recorded. A spike time ${T_i}$, which is the peak time of the neural spike, was also associated with the isolated neural spike $s_i^c\left( t \right)$.

### 4.3. Elimination of duplicated neural spikes

It is apparent from figure 1(c) that when a spike was fired by a neuron, it can be simultaneously sensed by multiple neighboring recording channels. This phenomenon is due to the close physical spacing between these channels, which causes the ionic current to be recorded by multiple adjacent channels, effectively leading to the recording of duplicated neural spikes. Generally speaking, the ionic current induced by a neural spike decreases inversely over the propagation distance, causing the recording channel closest to the active neuron to record largest signal strength (Ranck 1963, Chen *et al* 2014, Obien *et al* 2015, Mohammadi *et al* 2019b). In addition, since voltage signal propagation in an ionic solution is an electromagnetic transmission effect which propagates with a speed close to the speed of light, all channels record the same spike with no observable signal delays (Humphrey and Schmidt 1990, Jun *et al* 2017). This zero-delay property can be used as a criterion to identify duplicate neural spikes.

In this paper, the neural spike with the highest peak voltage was chosen to represent the entire duplicate set of neural spikes, as shown in figure 2(c). Other selection criteria, such as combining or averaging neural spikes, can also be used to select the representing neural spike; However, our experience indicates that combining these neural spikes did not result in better sorting accuracy (data not shown). Therefore, in this work we simply use the neural spike with the strongest signal to represent the entire group of duplicated neural spikes for simpler computation. Generally speaking, neural spikes with the highest peak intensities also have the best Signal-to-Noise Ratio, which typically results in good sorting accuracy (Mohammadi *et al* 2019a).

One major advantage of this duplicate spike elimination approach is to significantly reduce the number of neural spikes that need to be processed, hence reducing computation cost. Due to the fast signal propagation speed, only neural spikes with the same peak arrival time were considered to belong to the cluster of duplicate spikes. In addition, channel adjacency is an important factor to consider when determining whether neural spikes arriving the same time belong to the same neural spike fired by a neuron. If the neural spikes had similar temporal features but were measured by channels physically remote, these neural spikes were considered to be fired by two different neurons which happened to have the same temporal features. Pearson’s correlation was then used to determined temporal feature similarity between two neural spikes. The Pearson correlation coefficient ${r_{i,j}}$ between the two neural spikes $s_i^{{c_i}}\left( t \right)$ and $s_j^{{c_j}}\left( t \right)$ measured from channel and is

\begin{align*}{r_{ij}} = \frac{{{\text{Cov}}\left( {s_i^{{c_i}}\left( t \right),s_j^{{c_j}}\left( t \right)} \right)}}{{{\sigma _i}{\sigma _j}}}\end{align*}

where ${\sigma _i}$ and ${\sigma _j}$, the standard deviation of the and ${j^{{\text{th}}}}$ neural spikes, and is the covariance between two neural spikes. In this work, the two neural spikes are considered to belong be duplicates if ${r_{i,j}}$ is larger than ${r_{{\text{TH}}}}$ (${r_{{\text{TH}}}} = 0.6$ was used in the work). Once neural spikes with high correlations were identified, the neural spike with the highest peak signal was selected as the representing neural spike${\text{ }}\mathcal{S}_{\text{i}}^{\text{c}}\left( {\text{t}} \right)$ (${\text{c}}$ as the channel measuring the strongest neural spike). The remaining duplicated neural spikes were discarded since including these spikes in the classification process can lead to counting duplication.

### 4.4. PCA based feature extraction trained with initial spikes

Once the representing neural spike $\mathcal{S}_i^c\left( t \right)$ was determined, the neural spike was then sent to a dimension reduction algorithm for feature extraction. In addition, the representing neural spike $\mathcal{S}_i^c\left( t \right)$ was associated with a channel feature $c$, which was used to differentiate neural spikes measured from different areas of the neural probe.

PCA was used to extract features from the representing neural spike $\mathcal{S}_i^c\left( t \right)$. Traditional PCA analyzes the entire set of neural spikes to determine the principal components for feature extraction, but this approach is incompatible with real-time neural spike sorting. Since neural probes are mounted securely within the animal brain, temporal profiles of the recorded neural spikes do not change rapidly, but can slowly drift after hours of recording due to small movements of tissue. Based on this assumption, principal components ${f_n}\left( t \right)$ can be learned merely by using neural spikes measured during a short training period based on the standard PCA routine. Features of streamed-in neural spikes can be extracted using the estimated principal components. The $n$ th features $a_{n,i}^c{\text{ }}$ of a representing neural spike $\mathcal{S}_i^c\left( t \right)$ measured from channel $c$ can be calculated by

\begin{align*}{a_{n,i}} = \mathop \int \limits_{{t_s}}^{{t_e}} \mathcal{S}_i^c\left( t \right){ }{f_n}\left( t \right){\text{d}}t\end{align*}

where ${f_n}\left( t \right)$ is the $n$th principal component; ${t_s}$ and ${t_e}$ are the beginning and end time of the isolated neural spike epoch. In GEMsort, the channel is used as one of the features to separate neural spikes measured from different locations. The feature ${c_i}$ of the representing neural spike is ${x_i} = \left\{ {{c_i},{\text{ }}{a_{0,i}},{a_{1,i}},{a_{2,i}} \cdots } \right\}$. This channel feature $c$ is an important improvement to the original single channel algorithm to allow for the separation of clusters measured from different recording channels to avoid cluster overlaps, as shown in figure 2(d). Due to dimensional reduction of PCA, only the first few features of neural spikes ($n = 2$ for this work) are needed to be sent to the next step for clustering.

### 4.5. Graph network based neuron spike clustering

Neural spikes fired by the same neuron have similar temporal features, which allows cluster formation in the feature space. Unsupervised learning techniques can then be used to classify these clusters. In GEMsort, instead of examining the entire set of recorded neural spikes, a graph network approach allows for the examination of neural spikes one at a time, and this individual spike examination allows for rapid sorting. In addition, the algorithm learns the neural clustering distribution on the fly through forming, deleting or moving these surrogated clusters constructed by nodes and edges.

GEMsort is based on our previously demonstrated real-time graph-based spike classification for single channel recording (known as Enhanced Growing Neural Gas classification) modified with duplicated neural spike elimination and additional channel features. The details of this single-channel classification algorithm can be found in (Mohammadi *et al* 2019a), and a brief description of that method is provided below. In EGNG, neural clustering can be learned by nodes forming clusters in the feature space. When a neural spike is measured, the Euclidean distance between the spike features and the nodes are compared and the neural spike is classified to the cluster group containing the node closest to the neural spike. Suppose the features of the representing neural spike $\mathcal{S}_i^c\left( t \right)$ is ${x_i} = \left\{ {{c_i},{\text{ }}{a_{0,i}},{a_{1,i}},{a_{2,i}} \cdots } \right\}$ and the node is denoted as ${N_j}$, the Euclidean distance between the representing neural spike feature $x_i^c$ and the ${j^{{\text{th}}}}$ node ${w_j}$ is

\begin{align*}{d_{i,j}} = ||{x_i} - {w_j}||.\end{align*}

Edges connecting the nodes to form clusters can be created by identifying the closest node and the second closest node ${W_1} = {\text{argmi}}{{\text{n}}_{{w_j} \in N\left\{ {{W_1}} \right\}}}\left( {{d_j}} \right)$ to the neural spike. If the two closest nodes have not been connected by an edge, a new edge is formed to connect the two nodes. Through these edge connections, clusters can be formed for classification. In addition, if the two nodes connecting an edge have not been identified as closest nodes for an extended period, the edge can be deleted to indicate the dissociation of the two nodes, eventually leading to cluster dissociation.

This node and edge approach allows the clusters to adapt and learn the neural cluster distribution by moving the closest node ${W_1}$ and its neighboring nodes ($j \ne 1$) closer to the neural spike features based on the moving rates (${e_{S1}}$ and ${e_{{\text{nbr}}}}$).

\begin{align*}{W_1} \leftarrow {W_1} + {e_{S1}}\left( {{x_i} - {W_1}} \right)\end{align*}

\begin{align*}{W_j} \leftarrow {W_j} + {e_{{\text{nbr}}}}\left( {{x_i} - {W_j}} \right).\end{align*}

New nodes can be procedurally added to expand the clusters when neural spike features repeatedly appear in an area, and unused nodes can be deleted when neural spikes are no longer being recorded in certain regions in the feature space. Based on this dynamic clustering, clusters can dynamically adjust, formed and deleted when the neural clustering distribution is slowly changing during long recording sessions.

The pseudo-code of this graph-based clustering approach can be found in the supplementary information.

### 4.6. Rapid neural spike clustering through identifying the closest cluster

With this graph approach, the neural clustering distribution can be adaptively learned by the nodes and edges, retaining previously recorded neural spikes in the system memory to inform that future clustering is not needed. Classification of a neural spike can be rapidly estimated through determining the graph cluster containing the closest node ${W_1}$ to the neural spike. The output of GEM spike sorting contains the neuron cluster number, the peak neural spike firing time and the channel feature. This information can be used for further neural data analysis. For instance, a firing time histogram against the neuron cluster number can be constructed based on this sorting outcome.

The employed parameters for the GEMsort algorithm are shown in table 4.

### 4.7. Program code and data availability

The python code of the GEMsort spike sorting package is available and published at https://github.com/Zeinab-Mohammadi/GEMsort. The GEMsort spike sorting package can be used to sort both the synthetic 16 channel data under neuron configurations and timing scenarios and the neural data measured by a Neuropixels probe. The software package also includes codes to produce the synthetic multichannel datasets for GEMsort. In addition, the raw Neuropixels data used in this paper to compare sorting results between GEMsort and Kilosort are publicly available at https://ndownloader.figshare.com/files/37854918.

## Data Availability

The data that support the findings of this study are openly available at the following URL/DOI: https://ndownloader.figshare.com/files/37854918.
